# Care pathways, prescribing practices and treatment outcomes in major depressive disorder and treatment-resistant depression: retrospective, population-based cohort study

**DOI:** 10.1192/bjo.2023.627

**Published:** 2024-01-19

**Authors:** Sofia Pappa, Moulesh Shah, Sophie Young, Tazneem Anwar, Timothy Ming

**Affiliations:** Department of Brain Sciences, Faculty of Medicine, Imperial College, London, UK; and West London NHS Trust, London, UK; Imperial College Health Partners, London, UK; Janssen Cilag Ltd, High Wycombe, UK

**Keywords:** Major depressive disorder, treatment-resistant depression, real-world data, treatment pathway

## Abstract

**Background:**

Despite the availability of effective therapies, many patients with major depressive disorder (MDD) develop treatment-resistant depression (TRD).

**Aims:**

To evaluate and compare prescribing patterns, contact with specialist services and treatment outcomes in patients with MDD and TRD.

**Method:**

This was a retrospective analysis of linked primary and secondary care National Health Service data in the north-west London Discover-NOW data-set. Eligible patients were adults who had diagnostic codes for depression and had been prescribed at least one antidepressant between 2015 and 2020.

**Results:**

A total of 110 406 patients were included, comprising 101 333 (92%) with MDD and 9073 (8%) with TRD. Patients with TRD had significantly higher risks of suicidal behaviour and comorbidities such as anxiety, asthma, and alcohol or substance misuse (all *P* < 0.0001). Citalopram, sertraline, fluoxetine and mirtazapine accounted for 83% of MDD and 71% of TRD prescriptions. Use of antidepressant switching (1% MDD, 7% TRD) and combination therapy (1%, 5%) was rare, whereas augmentation occurred more frequently in the TRD group (4%, 35%). Remission was recorded in 42 348 (42%) patients with MDD and 1188 (13%) with TRD (*P* < 0.0001), whereas relapse was seen in 20 970 (21%) and 4923 (54%), respectively (*P* < 0.0001). Mean times from diagnosis to first contact with mental health services were 38.9 (s.d. 33.6) months for MDD and 41.5 (s.d. 32.0) months for TRD (*P* < 0.0001).

**Conclusions:**

There appears to be a considerable difference between treatment guidelines for depression and TRD and the reality of clinical practice. Long-term treatment with single antidepressants, poor remission, and high relapse rates among patients in primary care highlight the need to optimise treatment pathways and access to newer therapies.

Major depressive disorder (MDD) is a leading cause of global disability. It is estimated to affect 185 million people worldwide, including nearly 2.3 million in the UK.^1^ Treatment-resistant depression (TRD) is a growing area of interest among researchers and mental health professionals. Typically, it is operationally defined as MDD that has not responded to two lines of oral antidepressants used for a sufficient length of time at adequate doses within a single episode.^2,3^ It is suggested that 10‒30% of patients with MDD have TRD,^4^ although rates vary widely.^3,5–7^ Irrespective, the burden on individuals and healthcare services is considerable, and capacity within specialist mental health services in the UK is often insufficient to meet demand.^8^

Treatment guidelines for depression all support early initiation of appropriate pharmaceutical and/or psychological (including cognitive–behavioural) therapies with careful monitoring of outcomes.^9–11^ After initial treatment, stepwise treatment augmentation and/or specialist interventions (e.g. electroconvulsive therapy or transcranial magnetic stimulation) are recommended dependent on clinical response, treatment history and tolerability of potential side-effects,^9–12^ although, again, limited capacity may hinder the full use of options. However, a range of factors, including individual patient characteristics, symptoms, course and combined comorbidities, are thought to contribute to the development of TRD and can pose difficulties in individualising therapy or achieving effectiveness.^13,14^ Rush and colleagues, for example, showed that patients who did not respond to two lines of treatment had a greater illness burden (depression chronicity and psychiatric or general medical comorbidities), a decreased likelihood of achieving remission and an increased risk of relapse.^4^ Nevertheless, prescribing of antidepressants has been progressively rising in the UK,^15^ and they are sometimes favoured over non-pharmacological therapies;^16^ moreover, many lines of antidepressants with similar mechanisms of action and efficacy are being used in primary care before referral to secondary services.^16^ Some patients with TRD are reported to have tried up to 12 antidepressants and waited 10 years before they are seen at specialist centres.^17,18^

A better understanding of current practices and treatment pathways for TRD is needed in order to improve patient outcomes and lessen overall disease burden. The aim of this study was to use linked real-world primary and secondary healthcare data to evaluate and compare characteristics, care pathways, prescribing patterns and treatment outcomes for patients with MDD and TRD.

## Method

### Study design and population

This was a retrospective analysis of treatment outcomes for patients in north-west London (NWL) with MDD and TRD. Eligible patients were adults (≥18 years old) who were registered with a general practitioner (GP) in NWL, had a diagnosis of depression (i.e. were assigned an ICD-10 or Read code [version 2] for depression) made before or during the study period, and had received a prescription for an antidepressant during the study period of 1 January 2015 to 31 December 2020. Exclusion criteria were ICD-10 or Read codes for bipolar, dementia, mania, psychosis, or schizophrenia and schizoaffective disorders recorded from 2010 onwards.

### Data warehouse and the Discover-NOW platform

In this observational, population-based study, we used data from the Discover-NOW Health Data Research Hub for Real World Evidence (Discover), the access platform for the longitudinal Whole Systems Integrated Care (WSIC) database, which is hosted by Imperial College Health Partners.^19^ WSIC contains linked coded primary, acute, mental health, community health and social care records that are collected as part of daily record keeping. The data-set is fed by more than 400 provider organisations, including 360 GP practices, two mental health services, two community trusts and all acute providers in NWL, and covers more than 2.5 million patients. Furthermore, it allows access to accurate commissioners’ costs at the patient level, enabling evaluation of direct costs associated with healthcare use throughout treatment pathways.

Discover deidentifies data to meet the minimisation standards of the Information Standards Boards of NHS Digital, providing only information on demographics, medical histories, consultation notes, test results and prescriptions. The NWL Data Access Committee considers requests for access and controls how data are accessed and used. The application for the data-set used in this study was approved in September 2020 and again in April 2021. The study sponsor did not have access to patient-level data.

### Data collection

The study index date for each patient was the first or earliest antidepressant prescription from 1 January 2015 onwards, after which patients were followed up until the end of the study period. The prescription date was used to ensure capture of any instances in which a code for depression was recorded after antidepressant prescription.

Data were collected on age, gender, body mass index, ethnicity, geographical location and antidepressant prescriptions. The antidepressants of interest are shown in Supplementary Table 1 available at https://doi.org/10.1192/bjo.2023.627. Information was also obtained on comorbidities, concomitant medications and treatment augmentation drugs of interest (Supplementary Table 3). Where available in the primary care records, data were collected on self-harm, suicide attempts and suicidal ideation. Data on access to the Improving Access to Psychological Therapies services and related outcomes are not captured in the Discover-Now database and were therefore not included here.

The data-set was interrogated using SQL scripts that linked queries across data tables through a patient's common key. The model had previously been used to interrogate a Clinical Practice Research Datalink data-set, and this was taken as validation of the model.^16^ Minor adjustments were made to definitions to tailor the model to the Discover data-set.

Extracted outputs were downloaded to a secure server and aggregated in compliance with the information governance suppression rule, in which values of 0‒4 are annotated as <5.

### Definitions

TRD was defined as no response after at least two sequences of different oral antidepressant therapies with adequate dose and duration within an MDD episode.

Outcomes were derived by observing predefined treatment dynamics; therefore, their interpretation is associated with significant limitations, as described in the Discussion. In the absence of established outcomes using prescription data, outcomes were defined incorporating extensive clinical input and considering the typical prescription length of 30 days.

Treatment response was defined as prescription of a single treatment sequence for ≥90 days with no change other than dose. Remission was indicated by response of at least 180 days or no antidepressant prescription in the 60 days following the end of a sequence of ≥90 days. Relapse was signified by prescription of an antidepressant treatment sequence within 60‒180 days after the end of a previous sequence that had resulted in a response.

To account for delays in clinical practice (e.g. late start of a prescription, drug out of stock or delay in obtaining a repeat prescription), treatment was classified as follows. Switching was indicated by at least one antidepressant being stopped and at least one replacement drug being introduced within 60‒180 days. Combination prescriptions were those to which an antidepressant was added for at least 45 days when compared with the previous sequence. Pharmaceutical treatment augmentation was signalled by the addition of lithium, an antipsychotic, thyroxine, tri-iodothyronine or an anticonvulsant to a treatment sequence for at least 45 days. Patients classified as having treatment failure were those with lack of treatment response or who switched, started combination therapy or started augmented treatment. Time from diagnosis to referral was defined as the duration (in days) from a depression code being recorded to a code for first referral or contact with secondary mental health services being recorded.

Contact with secondary mental health services was identified by a Read code for referral in primary care or by a recorded contact with the service to include patients referred via a source other than a primary care practitioner. These included attendance at an accident and emergency department, in-patient or out-patient care by specialist mental health services, and use of home treatment, a crisis intervention team, liaison services or community services.

### Subgroups

As well as comparing patients with MDD and those with TRD, we further divided the TRD subgroup into patients who had received two, three or more, or four or more antidepressant prescriptions. In addition, we considered patients who had contact with secondary mental health services as a subgroup.

### Study objectives

Our hypothesis was that patients with TRD would have a considerably higher burden in terms of treatment outcomes, suicidality, and psychiatric and physical comorbidities than patients with MDD, and that there would be significant treatment gaps and delays in referring to and accessing primary and/or secondary services compared with guidelines.

Hence, our main objectives were to compare time from diagnosis to first contact with secondary mental health services, time to treatment response or end of the study (whichever occurred first) and which antidepressants are prescribed in primary care for patients with MDD versus those with TRD.

In addition, we compared response, remission and relapse rates by number of lines of treatment (≤2 *v.* ≥3 and ≥4 lines of therapy), presence of self-harm or suicidal behaviour, presence of comorbidities, and use of patient-reported measures and assessments (the brief Patient Health Questionnaire [PHQ-9], the Generalized Anxiety Disorder Assessment [GAD-7] and the Alcohol Use Disorders Identification Test Consumption [AUDITC]) between the MDD cohort and the TRD subgroup.

### Statistical analysis

Data were generated by descriptive analysis and inferential statistics. Continuous variables in descriptive analyses are expressed as mean and standard deviation, or median and range. For categorical variables, counts and proportions are presented. Comparisons between groups were performed with the χ^2^ test (d.f. = 1) for categorial variables or Wilcoxon rank sum test with continuity correction for numerical variables. Significance is denoted by *P*-values less than 0.05.

## Results

**Fig. 1:**
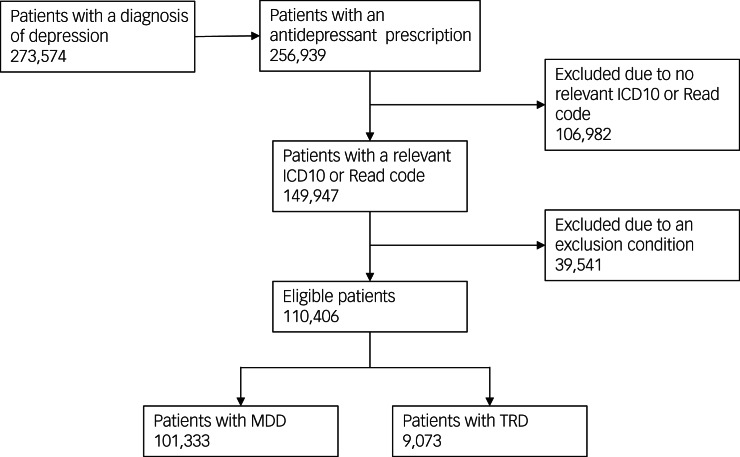
Study profile. MDD, major depressive disorder; TRD, treatment-resistant depression.

From January 2015 to December 2020, 273 574 patients had a recorded diagnosis of depression, and 256 939 patients were given prescriptions for antidepressants. Of these, 110 406 (74%) met the inclusion criteria, with 101 333 (92%) being classified as having MDD and 9073 (8%) as having TRD (Fig. 1). Around half of the overall cohort entered the study in 2015 owing to patients having pre-existing codes for depression (Supplementary Fig. 1 and Table 3).

The mean time from index date to the end of the study was 49.9 (s.d. 21.8) months for the overall cohort (median 58 months, range 0‒71). In the MDD group, the mean time in the study was 49.2 (s.d. 22.0) months (median 56 months, range 0‒71), compared with 59.1 (s.d. 16.0) in the TRD group (median 68 months, range 0‒71). For the TRD subgroups, the values were 60.9 (s.d. 13.9) months for who had received three or more lines of therapy and 62.5 (s.d. 12.3) months for those who had received four or more lines (median 68 months, range 3‒71; and median 69 months, range 5‒71, respectively). For patients who had contact with secondary mental health services, the mean time in the study was 51.9 (s.d. 21.3) months (median 61 months, range 0‒71).

### Patients’ characteristics and comorbidities

Most patients were aged between 20 and 59 years in the MDD and TRD groups, and two-thirds of the cohort were female (Table 1). More than half of patients were White, but socioeconomic status was similar across the cohort (Supplementary Table 4). Body mass index did not differ between MDD and TRD patients. Employment status was not well recorded, but among those with data, most were unemployed, and this was more so in the TRD group.

**Table 1:** Study population characteristics

Characteristic	Whole cohort (*n* = 110 406)	Subgroups		
		MDD (*n* = 101 333)	TRD (*n* = 9073)	*P*-value for MDD *v.* TRD
Age (years)	44.1 (16.4)	44.1 (s.d. 16.5)	43.4 (s.d. 15.7)	0.21
Sex^a^				0.078
Male	40 918 (37%)	37 690 (37%)	3228 (36%)	–
Female	68 514 (62%)	62 794 (62%)	5720 (63%)	–
BMI (kg/m^2^)	27.6 (s.d. 26.6)	27.4 (s.d. 6.6)	27.7 (s.d. 6.6)	0.16
Ethnicity				
White	59 233 (54%)	54 172 (53%)	5051 (56%)	<0.0001
Black	8173 (7%)	7630 (8%)	543 (6%)	<0.0001
Asian	19 993 (18%)	18 344 (18%)	1589 (18%)	0.16
Mixed	3814 (21%)	3471 (3%)	343 (4%)	0.93
Other	9523 (9%)	8680 (9%)	843 (9%)	0.063
Unknown	9740 (9%)	9036 (9%)	704 (8%)	<0.0001
Employment status^b^				
Unemployed	9369 (9%)	7964 (8%)	1405 (15%)	N/A^b^
Employed	5585 (5%)	5099 (5%)	486 (5%)	N/A^b^
Retired	1966 (2%)	1764 (2%)	202 (2%)	N/A^b^
Other	50 (<1%)	49 (<1%)	1 (<1%)	N/A^b^
Smoking status				
Non-smoker	50 056 (45%)	46 033 (45%)	4023 (44%)	N/A^b^
Smoker	31 620 (29%)	28 556 (28%)	3064 (34%)	N/A^b^
Ex-smoker	15 563 (14%)	14 316 (14%)	1247 (14%)	N/A^b^
Unknown	13 167 (12%)	12 428 (12%)	739 (8%)	N/A^b^
Comorbidities				
Alcohol/substance misuse	11 787 (11%)	10 256 (10%)	1531 (17%)	<0.0001
Anxiety	30 629 (28%)	26 918 (27%)	3711 (41%)	<0.0001
Asthma	13 515 (12%)	12 135 (12%)	1380 (15%)	<0.0001
COPD	5121 (5%)	4614 (5%)	507 (6%)	<0.0001
Diabetes	13 204 (12%)	12 051 (12%)	1153 (13%)	<0.022
Epilepsy	1288 (1%)	994 (1%)	294 (3%)	<0.0001
Ischaemic heart disease	6074 (6%)	5548 (5%)	526 (6%)	<0.21
PTSD	295 (<1%)	222 (<1%)	73 (1%)	<0.0001
Sleep disturbance^c^	2220 (2%)	1937 (2%)	283 (3%)	<0.0001

BMI, body mass index; MDD, major depressive disorder; TRD, treatment-resistance depression; COPD, chronic obstructive pulmonary disease; PTSD, post-traumatic stress disorder.

a. Numbers do not sum to total as not all patients chose either of these categories.

b. Available for only 23% of the cohort.

c. Coded for only 10% of the cohort.

Patients with MDD had fewer comorbidities than those with TRD (mean 0.74 [s.d. 0.9] *v.* 1.04 [s.d. 1.0]) and the difference extended with increasing lines of therapy (1.13 [s.d. 1.0] with three or more lines and 1.20 [0.9] with four or more lines). Anxiety was most commonly reported (Table 1). Frequency of anxiety was around 1.5 times higher in patients with TRD than in those with MDD (1451 [48%] of 9073 *v.* 26 918 [27%] of 101 333), rising to more than double in the TRD subgroup of patients who received four or more lines of therapy (481 [57%] of 845). Asthma and alcohol or substance misuse were the next most frequent comorbidities overall and, respectively, were seen roughly 1.2 and 1.7 times more often in the TRD group than in the MDD group (Table 1).

A little more than a quarter of patients (28%) were classified as smokers during the study period. The rate of smoking was lower in the MDD group than in the TRD group (28% *v.* 34%) and the group of patients in contact with secondary mental health services (35%).

### Duration of depression and treatment

**Fig. 2:**
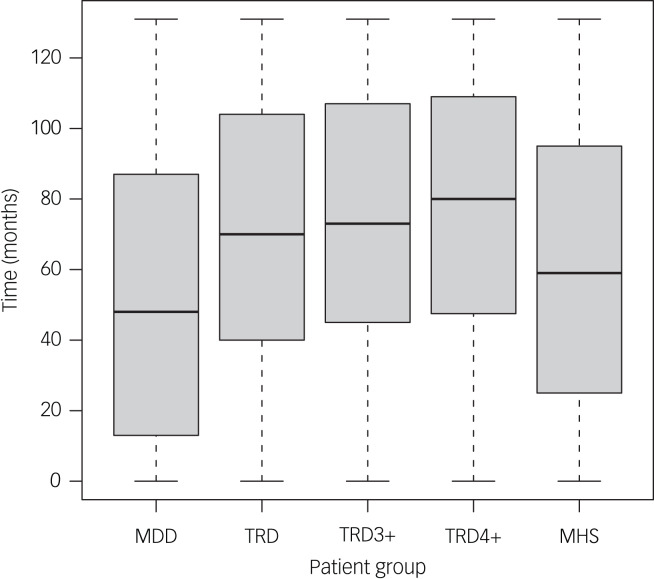
Mean duration of depression. MDD, major depressive disorder; MHS, contact with secondary mental health services; TRD, treatment-resistant depression; TRD3+, three or more lines of treatment for depression; TRD4+, four or more lines of treatment for depression. *P < 0.0001 *v.* MDD. The dark lines represent statistically significant findings (*p* ≤ 0.05).

The mean duration of depression before response or study end (whichever occurred first) was 52.8 (s.d. 41.7) months in the MDD group and 70.8 (s.d. 37.8) in the TRD group (*P* < 0.0001; 74.1 [s.d. 36.8] with more than three lines of therapy and 77.3 [s.d. 35.8] with more than four lines of therapy; Fig. 2). For those patients who had contact with secondary mental health services, the mean duration of depression was 60.7 (s.d. 40.3) months.

Fewer than a quarter of patients had contact with the secondary mental health services (25 893 [23%] of 110, 406), but the proportion in the TRD subgroup was more than double that than in the MDD group (4923 [54%] of 9073 *v.* 20 970 [21%] of 101 333). The average time from diagnosis to first contact with secondary mental health services did not differ substantially between the MDD and TRD groups (mean 38.9 [s.d. 33.6] months *v.* 41.5 [s.d. 32.0] months, *P* < 0.0001).

### Prescribing data

The numbers of prescriptions by type of medication are shown in Supplementary Table 5. The most frequently prescribed drug for MDD was citalopram (713 937 prescriptions, 35% usage), whereas that for TRD was mirtazapine (107 352 prescriptions, 29% usage). Fluoxetine and sertraline were used with similar frequencies in both groups. Together, these four drugs accounted for 83% of all prescriptions in the MDD group and 71% of prescriptions in the TRD group.

Across the duration of the study, the overall number of prescriptions was 2.4 million, most of which were in the MDD group (Table 2). In the TRD group, 2993 (33%) of 9073 patients had received three or more lines of antidepressants and 845 (9%) had received four or more lines. The mean number of prescriptions per patient over the study period was 22.0 (s.d. 36.2) in the whole cohort, 20.3 (s.d. 0.1) in the MDD group and 41.5 (s.d. 47.4) in the TRD group (42.9 [s.d. 45.9] and 44.3 [s.d. 40.7] in TRD patients who received more than three or more than four lines, respectively). Among all patients in contact with mental health services, the mean number of prescriptions per patient was 32.7 (s.d. 49.2).

**Table 2:** Numbers of antidepressant prescriptions during the study period

	Whole cohort	MDD	TRD	*P*-value MDD *v.* TRD	TRD3+	TRD4+	MHS
Overall prescriptions	2 428 120 (100%)	2 051 569 (84%)	376 551 (16%)	<0.0001	128 276 (5%)	37 432 (2%)	846 229 (35%)
Resumed prescriptions	1 155 001 (48%)	995 361 (49%)	159 640 (42%)	<0.0001	53 022 (41%)	16 826 (45%)	350 177 (41%)
Switch of prescriptions	35 387 (1%)	17 517 (1%)	17 870 (5%)	<0.0001	17 870 (5%)	2227 (5%)	16 630 (2%)
Combination prescriptions	47 128 (2%)	20 424 (1%)	26 704 (7%)	<0.0001	10 021 (8%)	2963 (8%)	28 074 (3%)
Augmentation prescriptions	6806 (6%)	3620 (4%)	3186 (35%)	<0.0001	1202 (40%)	404 (48%)	4350 (17%)

MDD, major depressive disorder; MHS, contact with secondary mental health services; TRD, treatment-resistant depression; TRD3+, three or more lines of treatment for depression; TRD4+, four or more lines of treatment for depression.

The mean times between the first and last prescriptions were 49.2 (s.d. 22.0) months and 59.1 (s.d. 16.0) months in the MDD and TRD groups, respectively. The mean duration of antidepressant treatment during the study period was 28.9 (s.d. 26.9) months in the MDD group and 51.0 (20.5) months in the TRD group (*P* < 0.0001).

Nearly half of all prescriptions were classified as resumed treatment (Table 2). The mean numbers of resumptions per patient were 13.9 (s.d. 16.2) overall, 13.4 (s.d. 16.1) in the MDD group and 18.5 (s.d. 16.2) in the TRD group (18.4 [s.d. 15.4] with three or more lines of therapy and 20.5 [s.d. 15.3] with four or more lines). For patients in contact with secondary mental health services, the mean was 16.3 (s.d. 17.3) resumed prescriptions. The number of switching and combination prescriptions was low overall, although patients with TRD received numerically (and, therefore, proportionately) more combination prescriptions than those with MDD. The mean number of combination prescriptions per patient was roughly 1.4 times lower for those with MDD that for those with TRD (4.5 [s.d. 13.4] *v.* 6.51 [s.d. 16.1]). The use of treatment augmentation prescriptions was numerically similar in the MDD and TRD groups (1.4 [s.d. 0.7] *v.* 1.5 [s.d. 0.7]) and hence proportionately much higher in the latter group (Table 2).

### Clinical outcomes: response, remission and relapse rates

The mean numbers of responses per patient were 1.0 (s.d. 0) for the MDD group and 1.6 (s.d. 0.7) for the TRD group, giving a significantly better rate in the latter group (30% *v.* 26%, *P* < 0.0001; Table 3). With three or more lines of treatment, the rate rose to 2.1 (s.d. 0.8), and with four lines or more it increased further to 2.7 (s.d. 1.0). Contact with secondary mental health services was associated with only a slight increase in the number of responses per patient compared with the MDD group (mean 1.1 [s.d. 0.2]). Relapse was recorded in 31% of patients in the MDD group and 53% of patients in the TRD group (*P* < 0.0001) but in only 22% of those in contact with secondary mental health services (Table 3). The mean rates of relapse were 1.4 (s.d. 0.7), 1.5 (s.d. 0.8) and 1.4 (s.d. 0.6) for the MDD group, TRD group, and patients in contact with secondary mental health services, respectively. The value was slightly higher for patients with TRD who received more than three (1.7 [s.d. 0.09]) or more than four (1.7 [s.d. 0.9]) lines of treatment. Remission was reported in 42% of patients in the MDD group compared with 13% in the TRD group (Table 3).

**Table 3:** Response, relapse, and remission rates

Patient group	Number of instances	Number of patients (percentage of cohort)
Response^a^		
MDD (*n* = 101 333)	26 686	26 686^b^ (26%)
TRD (*n* = 9073)	4259	2699 (30%)
MHS (*n* = 25 893)	8655	7728 (30%)
Relapse^c^		
MDD (*n* = 101 333)	44 189	31 074 (31%)
TRD (*n* = 9073)	7434	4842 (53%)
MHS (*n* = 25 893)	8296	5747 (22%)
Remission^d^		
MDD (*n* = 101 333)	42 348	42 348 (42%)
TRD (*n* = 9073)	1188	1188 (13%)
MHS (*n* = 25 893)	5438	5438 (21%)

MDD, major depressive disorder; MHS, contact with secondary mental health services; TRD, treatment-resistant depression.

a. Prescription of a single treatment sequence for ≥90 days with no treatment changes (excluding dose changes).

b. Matches number of instances because patients would have responded to first treatment and not received a second or responded to second to be defined as MDD.

c. Prescription of an antidepressant treatment sequence within 60–180 days after the end of a previous sequence that resulted in a response or prescription of a single treatment sequence for 180 days or longer without change.

d. No antidepressant prescription within 180 days of the end of the previous treatment sequence.

### Suicidal behaviours and self-harm

Few patients had recorded codes for suicidal ideation, suicide attempt and self-harm (7169 [6.5%] overall; Table 4). However, these behaviours were more likely to be reported in the TRD group than in the MDD group (mean number of records per patient 1.54 [s.d. 1.4] *v.* 1.38 [s.d. 0.9]). These values rose with increasing number of therapies in the TRD group (1.68 [s.d. 1.7] with three or more lines of therapy and 1.9 [s.d. 2.5] with four or more lines of therapy). Contact with secondary mental health services seemed to have little effect (mean number of reports per patient 1.4 [s.d. 1.1]).

**Table 4:** Numbers of reports of suicidal ideation and attempts and self-harm

Categories	Whole cohort (*n* = 110 406)	MDD (*n* = 101 333)	TRD (*n* = 9073)
Suicidal thoughts and ideation	6653 (6.0%)	5298 (5.9%)	1355 (14.9%)
Suicide attempt and attempted suicide NOS	290 (0.3%)	252 (0.3%)	38 (0.4%)
Intentional self-harm by unspecified means	225 (0.2%)	176 (0.2%)	49 (0.5%)
All	7169 (6.5%)	5727 (5.7%)	1442 (12.6%)

MDD, major depressive disorder; NOS, not otherwise specified; TRD, treatment-resistant depression.

### Use of assessment tools

The numbers of codes for use of the PHQ-9, GAD-7 and AUDITC tools to assess patients were low (31 215 [28%], 15 795 [14%] and 39 125 [35%] patients with results, respectively). In addition, use of the PHQ-9 was recorded only once or twice per patient; thus, it was not possible to perform a meaningful analysis for this tool. Use of GAD-7 classified most patients as having severe anxiety, with the proportions assigned this classification ranging from 36% of patients in the MDD group to 54% of patients with TRD who had received more than four lines of therapy. Of note, 6614 patients who had contact with secondary mental health services were assessed by GAD-7, but no score was recorded for 2786 (42%). Among the remaining 3828 patients with results, moderate anxiety was most frequent, followed by mild anxiety, no anxiety and severe anxiety (Supplementary Table 6). AUDITC results indicated that around 75% of patients were at low risk of alcohol misuse and only 3‒4% were at high risk in all groups.

## Discussion

This study included a large cohort comprising more than 100 000 patients with MDD and more than 9000 classified as having TRD. Furthermore, access to a comprehensive real-world data-set allowed a median follow-up of 5 years and enabled tracking of long-term treatment patterns and entire care pathways from primary to specialist secondary care. Our findings showed that patients with TRD had substantially higher risks of psychiatric and physical comorbidities, intentional self-harm and suicidal behaviour and poorer response, relapse and remission rates than patients with MDD. These gaps widened with increasing lines of treatment. Furthermore, there were considerable delays to referral and first contact with secondary care services despite the absence of much improved patient outcomes. In fact, only half of patients with TRD had contact with secondary mental health services, with an average time to first contact of around 3.5 years.

Around 8% of patients in our study were classified as having TRD. This percentage is far lower than the 30% prevalence reported in the STAR*D study^4^ but much closer to that estimated in the Global Burden of Disease Study and other real-world studies.^1,20^ The difference is most likely due to the implicit limitations of registry-based studies in accurately capturing all the changes in treatment dynamics but also the actual disparity in the frequency of medication changes (such as switching and augmentation) between routine clinical practice and clinical trials.^21^ A number of studies have shown that a high proportion of patients are maintained on the same medication for long periods of time following first- and second-line treatment despite lack of adequate response. For example, Wiles et al^18^ found that 72% of a cohort of still-depressed patients in primary care remained on the same medication at the same dose for a period of 12 months. In a study by Day et al^17^ of 178 patients with TRD, 47% and 51% continued an unsuccessful first or second antidepressant for more than 16 weeks, Furthermore, switching from the first or second trial drug did not occur for at least 1 year for 24% and 27%, respectively.

Within the National Health Service (NHS), most MDD diagnoses are initially recorded in primary care and treated by GPs, with regional guidelines usually advising a referral to secondary care mental health services and a psychiatrist only for those with complicating factors such as high risk or treatment resistance. Secondary care services provide both urgent and routine community and in-patient care, as well as specialist interventions such as electroconvulsive therapy. Currently, the model recommended by the National Institute for Health and Care Excellence for treating patients with depression in the UK is a stepped-care approach based on intensity, response and chronicity. For mild depression, psychoeducation, exercise, and talking therapies such as cognitive–behavioural therapy or counselling are usually offered. For more severe or persistent depression, antidepressants and evidence-based psychological treatments (i.e. cognitive–behavioural therapy, behavioural activation, interpersonal therapy or psychotherapy) should be considered before being offered high-intensity interventions and combination treatment. In-patient care and electroconvulsive therapy are reserved for emergency situations or complex cases.^9^

In the present study, remission was achieved in only 42% of patients in the MDD group and 13% in the TRD group; this was similar to the results reported by Heerlein et al in a 1 year study of 411 patients,^22^ where the remission rates were 16.7% at 6 months and 19.2% at 12 months. The relapse rate in our study was 31% in the MDD group and 53% in the TRD group. Likewise, over a 2 year follow-up period, Gillain and colleagues found that of 95 patients who achieved response or remission, 61% relapsed.^23^

The average number of prescriptions per patient was 22 overall, but the number in patients with TRD was twice that in those with MDD. Most antidepressants have an established efficacy in the treatment of depression,^24,25^ with little difference between them in terms of overall outcomes.^26^ The effects, however, are achieved by a variety of mechanisms, and patients might benefit from drug combinations.^27,28^ In our study, of 30 different antidepressants assessed, only four (citalopram, mirtazapine, fluoxetine and sertraline) accounted for 83% of prescriptions for patients with MDD and 71% of prescriptions for those with TRD. This finding is similar to that of Bogowicz et al, who reported that the ten most commonly prescribed antidepressants in 2018 accounted for 96.7% of all antidepressant usage.^15^ In addition, in accordance with previous studies, we found that approximately half of prescriptions were resumptions, whereas rates of switching and combination were low in both groups, and only augmentation occurred more frequently in the TRD group. Heerlein and colleagues, for example, showed that in 60% of patients they assessed, prescriptions had not changed over time despite the low remission rates. These practices, albeit common, are not in line with the UK national guidelines,^9^ which recommend considering changes in treatment (e.g. dose adjustment, switching, combination, augmentation) if no effect has been observed within 4‒6 weeks.

Reluctance to adopt more assertive practices may be partly due to a lack of available evidence and clear algorithms for pharmaceutical optimisation.^27–29^ Furthermore, several new promising treatments that have been approved specifically for the management of TRD over recent years, such as repetitive transcranial magnetic stimulation, vagal nerve stimulation, and NMDA-receptor antagonist compounds,^30^ or were in the pharmaceutical pipeline,^31^ were not available within specialist services at the time of the study. Therefore, treatment still relied mostly on antidepressants and psychological approaches even for patients with TRD. Despite more than a quarter of our TRD subgroup receiving more than four lines of therapy, the average wait for the first contact with secondary mental health services was around 3.5 years. These findings are in line with a recent pan-European study showing that many people with symptoms of depression are not able to access or benefit from usual treatments, are not followed up adequately, and cannot be referred to secondary care or specialist services when required, with only around 19% of people with an MDD diagnosis able to access secondary/psychiatric care.^32^

Thus, it appears pertinent to refocus clinical pathways and improve practice within primary care. Guidelines regarding earlier and easier access to secondary mental health services, more switching, combining and augmentation of treatments, and more specialist treatment need to be highlighted in primary care. Gaynes and colleagues found that if identical care was made available in primary and specialty settings, the same outcomes in terms of responses and remissions could be achieved.^33^ However, staff received support from a clinical research coordinator, the study was small, and patients in the two settings were similar, which is not reflective of all real-world populations. Bogowicz et al suggested that GPs’ knowledge of antidepressant guidelines and attitudes towards legacy prescribing should be explored.^15^ To this end, further research may be required to understand the barriers to the implementation of clinical depression guidelines within various NHS settings.

As expected, comorbidity rates were significantly higher in the TRD group compared with the MDD group and grew with increasing number of lines of therapy. Anxiety was the most common psychiatric comorbidity, affecting nearly one-third of all patients. Rates were much higher in the TRD group than in the MDD group, being almost doubled with four or more lines of treatment. Anxiety is a frequent comorbidity in depression and has been associated with both severity of disease and treatment resistance.^34,35^ Other important comorbidities included asthma, diabetes, and alcohol and substance misuse. Major depression is has been shown to be a notable risk factor for new-onset asthma in adults,^36,37^ and there is thought to be shared genetic liability between MDD and atopic diseases.^38^ The prevalence of diabetes in the study cohort was similar across subgroups and in line with previously reported rates.^39^ Finally, the co-occurrence of depression with smoking and alcohol/substance misuse, albeit not fully understood, is well established.^40^ A Swedish study involving nearly 3 million people in the general population indicated that the risk of alcohol and substance misuse was raised 4.5 times in people with generalised anxiety and depression disorders.^41^ Although this comorbidity is likely to have been under-recorded overall in the data-set we used, it was significantly more likely to occur in individuals with TRD in our sample.

Despite the overall considerable burden of MDD – and, particularly, TRD – our study emphasises the fact that treatment gaps between current and best practice and divergence from evidence-based international recommendations remain substantial. Notwithstanding some level of regional variations in care provision and treatment guidelines, this appears to be a consistent challenge across most parts of the UK and Europe. For example, contemporary international standards and clinical depression guidelines highlight the value of altering the course of therapy, combining therapies and/or considering timely specialist interventions, but clinical practice remains suboptimal. The recently published Value of Treatment mission study from the European Brain Council put forward a set of recommendations to address and minimise gaps relating to increased capacity, creation of more specialist mood disorders services, bespoke training programmes for GPs to achieve mental health expertise, and reforms to ensure early referrals and easy access to secondary services and specialist treatment.^32^

### Strengths and limitations

This study had considerable strengths, including the use of longitudinal data obtained from a large deidentified data-set via the Discover platform.^19^ WSIC is one of the largest data warehouses in Europe, and data linkage allows tracking of treatment dynamics, use of different parts of the healthcare system, comorbidities and medications. An additional advantage was that it was possible to assess TRD patients by number of lines of therapy. The point prevalence of MDD at the time of data extraction was 4375 per 100 000 patients, and that of TRD was 392 per 100 000 patients in NWL. Therefore, our sample size was highly representative.

The study, however, had several limitations. Real-world data are subject to coding and reporting errors and gaps, and capture was limited to NHS data. Therefore, records might have been missing for a variety of reasons. First, patients who received non-NHS private or residential care or were in groups vulnerable to mental health issues, such as prisoners, illegal immigrants and the homeless, could not be captured or were likely to be poorly captured. Second, some important aspects of the overall management of depression, such as suicidal behaviours and repeat testing of severity with PHQ-9 or other validated scales, patients who remain on an ineffective treatment without reporting lack of response (i.e. not reaching our threshold for TRD) and use of psychological therapies were not captured separately or well represented in the data. Third, prescriptions in secondary care are likely to be underrepresented, as they are recorded only if they incur high costs. However, this is unlikely to have greatly biased the results, as most prescribing for MDD occurs in the primary care setting. Fourth, the subgroup of patients in contact with secondary mental health services could not generally be assessed by diagnosis. The findings for this subgroup, however, tended to fall between those for MDD and TRD, potentially indicating that they were quite strongly driven by patients with TRD. Fifth, we were unable to capture data on specialist treatments available exclusively in secondary care, such as electroconvulsive therapy. Sixth, without strong data on the use of instruments, such as the PHQ-9, Hamilton Rating Scale for Depression and Montgomery–Åsberg Depression Rating Scale, we had to derive response, remission and relapse statuses through prescription data. As study-specific algorithms were developed to identify outcomes of interest, definitions used may have varied between studies using the same data. For example, definitions using different cut-off time points would have resulted in different outcomes. Furthermore, prescription data did not capture whether the prescription had been dispensed or consumed, a limitation of most electronic medical record databases. For the purposes of this study, it was assumed that all prescriptions written were dispensed on the same day and consumed according to the prescribing information. Improved recording of scores might be helpful in the evaluation of clinical outcomes in the future. Finally, the particular study design did not allow assessment of causality, and it was only possible to explore correlations. Therefore, cautious interpretation of findings is advisable. Adding to this, the two groups were quite dissimilar (including the large difference in sample size between the MDD and TRD cohorts), posing some degree of challenge when trying to make comparisons, although statistical tests may partly account for this. We used the Wilcoxon rank sum test in our inferential statistical analysis. A propensity score-matching method might have allowed for better comparison; however, it would not cover the entire population and thus would not capture all resource usage. Given the nature of this study, we decided that reflecting overall patterns would be more useful. Matching of groups might be possible as the Discover data-set grows in size.

### Clinical implications

This real-world population-based study allowed exploration of care received in primary and secondary NHS services in a large patient cohort and over a substantial period of time. Patients with MDD are receiving high numbers of prescriptions in primary care with few changes over several years despite low response and remission rates and without contact with secondary mental health services. Outcomes are even worse for patients with TRD, for whom the number of prescriptions per patient are at least doubled while the remission rate is decreased by around a third. The burden is also substantially higher for the TRD group regarding comorbidities such as anxiety, asthma and alcohol/substance misuse, as well as self-harm and suicidal behaviours. Furthermore, our findings suggest that long delays are not conducive to secondary care improving outcomes. Hence, along with optimising use of existing evidence on the use of available treatments and lessening variation from guidelines, the development of stratified care pathways ensuring timely access to new therapies and specialist services where needed might improve patient outcomes.^42^ In addition, reaching consensus on definitions and improved recording of routine data would be a benefit in future real-world studies to provide insight not available from clinical trials.

## Supporting information

Pappa et al. supplementary material Pappa et al. supplementary material

## Data Availability

The data that support the findings of this study are available from Imperial College Health Partners, but restrictions apply to availability as they were used under approval for the current study and are not publicly available. Researchers wishing to access WSIC/Discover-NOW data can apply to Imperial College Health Partners.
