# Supplementary material for: Care pathways, prescribing practices and treatment outcomes in major depressive disorder and treatment-resistant depression: retrospective, population-based cohort study
Source: BJPsych Open. 2024 Jan 19;10(1):e32. doi: 10.1192/bjo.2023.627 (PMC10897686; doi:10.1192/bjo.2023.627)
Supplement: Pappa et al. supplementary material [file S2056472423006270sup001.pdf]

Supplementary material

Care pathways, prescribing practices, and treatment outcomes in treatment-resistant depression: a population-based retrospective analysis of real-world data

Table A1: Medications of interest

SSRIs:

- Citalopram,
- Escitalopram,
- Fluoxetine,
- Fluvoxamine,
- Paroxetine,
- Sertraline.

Selective Norepinephrine Reuptake Inhibitors:

- Duloxetine,
- Venlafaxine.
- Desvenlafaxine

Tricyclic ADs:

- Amitriptyline (only if 100mg and over),
- Amoxapine,
- Butriptyline,
- Clomipramine,
- Desipramine,
- Dosulepin,
- Doxepin,
- Imipramine,
- Iprindole,
- Lofepramine,
- Nortriptyline,
- Protriptyline,
- Trimipramine,
- Viloxazine.

Tetracyclic ADs:

- Maprotiline,
- Mianserin,
- Mirtazapine,
- Trazodone (only if 150mg and over).

MAOIs:

- Iproniazid,
- Isocarboxazid,
- Moclobemide,
- Phenelzine,
- Tranylcypromine.

Other ADs:

- Agomelatine,
- Nefazodone,
- Reboxetine,
- Vortioxetine,

- Bupropion.

Table A2: Concomitant and augmentation medications

Antipsychotics

First generation

- Chlorpromazine
- Haloperidol
- Perphenazine
- Trifluoperazine

Second generation

- Aripiprazole
- Lurasidone
- Olanzapine
- Paliperidone
- Quetiapine
- Risperidone

Mood stabilisers/ anticonvulsants

- Lithium
- Sodium valproate (in the absence of an epilepsy diagnosis)
- Carbamazepine (in the absence of an epilepsy diagnosis
- Lamotrigine (in the absence of an epilepsy diagnosis)

Other

- Thyroxine T3
- Buspirone

Table A3: Study entry by diagnosis and calendar year

Calendar year	MDD (n=101,333)	TRD (n=9,073)	All patients (n=110,406)
2015	46,637 (46.0%)	5,936 (65.4%)	52,573 (47.6%)
2016	13,761 (13.6%)	1,214 (13.4%)	14,975 (13.6%)
2017	11,926 (11.8%)	875 (9.6%)	12,801 (11.6%)
2018	10,802 (10.7%)	594 (6.5%)	11,396 (10.3%)
2019	10,315 (10.2%)	354 (3.9%)	10,669 (9.7%)
2020	7,892 (7.8%)	100 (1.1%)	7,992 (7.2%)

Abbreviations: MDD, major depressive disorder; TRD, treatment-resistant depressions.

Figure A1: Rate of entry into the study by diagnosis and calendar year
Values in 2015 are much higher than in other years due to inclusion of patients with pre-existing diagnosis of depression. Abbreviations: MDD, major depressive disorder; TRD, treatment-resistant depressions.

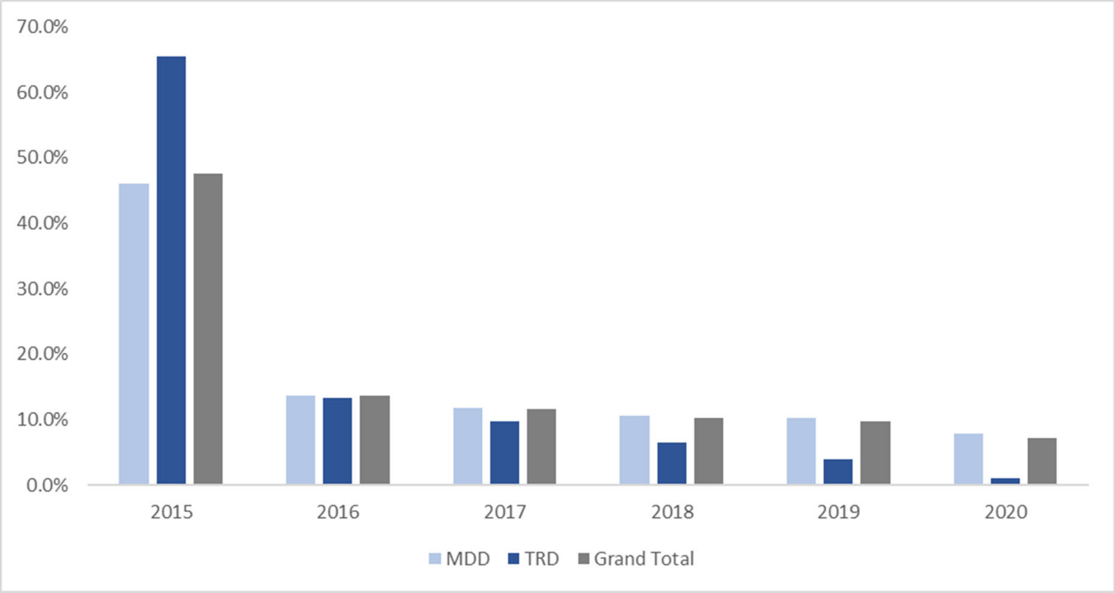

Table A4: Index of Multiple Deprivation score for patient cohort

IMD Score	MDD (n=101,333)	TRD (n=9,073)	TRD 3+ (n=2,993)	TRD 4+ (n=845)	Patients in contact with MH services (n=25,893)
UNKNOWN	5,505 (5%)	487 (5%)	153 (5%)	43 (5%)	1,300 (5%)
1	6,091 (6%)	504 (6%)	144 (5%)	37 (4%)	1722 (7%)
2	12,240 (12%)	1,096 (12%)	333 (11%)	98 (12%)	3407 (13%)
3	18,546 (18%)	1,774 (20%)	604 (20%)	163 (19%)	5253 (20%)
4	14,268 (14%)	1,343 (15%)	442 (15%)	124 (15%)	3748 (14%)
5	12,639 (12%)	1,108 (12%)	376 (13%)	117 (14%)	3283 (13%)
6	11,592 (11%)	983 (11%)	315 (11%)	80 (9%)	2861 (11%)
7	7,955 (8%)	688 (8%)	241 (8%)	85 (10%)	1826 (7%)
8	5,399 (5%)	441 (5%)	154 (5%)	44 (5%)	1147 (4%)
9	4,438 (4%)	388 (4%)	135 (5%)	27 (3%)	875 (3%)
10	2,660 (3%)	261 (3%)	96 (3%)	27 (3%)	471 (2%)

Abbreviations: MDD, major depressive disorder; MHS, contact with secondary mental health services; TRD, treatment-resistant depressions; TRD3+, three or more lines of treatment for depression; TRD4+, four or more lines of treatment for depression.

Table A5: Medications of interest, by diagnosis

BNF chemical substance	Number of prescriptions		
	**Whole cohort (n=2,428,120)**	**MDD (n=2,051,569)**	**TRD (n=376,551)**
Citalopram hydrobromide	779,191 (32%)	713,937 (35%)	65,254 (17%)
Mirtazapine	546,381 (23)	439,029 (21%)	107,352 (29%)
Fluoxetine hydrochloride	365,431 (15%)	317,390 (15%)	48,041 (13%)
Sertraline hydrochloride	285,574 (12)	240,179 (12%)	45,395 (12%)
Venlafaxine	139,398 (6%)	99,501 (5%)	39,897 (11%)
Duloxetine hydrochloride	83,447 (3%)	60,495 (3%)	22,952 (6%)
Paroxetine hydrochloride	80,003 (3%)	69,116 (3%)	10,887 (3%)
Escitalopram	63,803 (3%)	52,725 (3%)	11,078 (3%)
Dosulepin hydrochloride	19,506 (1%)	17,252 (1%)	2,254 (1%)
Nortriptyline	13,417 (1%)	9,163 (<1%)	4,254(1%)
Trazodone	9,626 (<1%)	7,119 (<1%)	2,507 (1%)
Clomipramine hydrochloride	8,230 (<1%)	5,632 (<1%)	2,598 (1%)
Vortioxetine	6,695 (<1%)	2,052 (<1%)	4,643 (1%)
Imipramine hydrochloride	4,800 (<1%)	3,742 (<1%)	1,058 (<1%)
Lofepramine hydrochloride	4,796 (<1%)	3,140 (<1%)	1,656 (<1%)
Bupropion hydrochloride	4,768 (<1%)	2,996 (<1%)	1,772 (<1%)
Trimipramine maleate	3,173 (<1%)	2,453 (<1%)	720 (<1%)
Agomelatine	2,886 (<1%)	1,242 (<1%)	1,644 (<1%)
Citalopram hydrochloride	1,708 (<1%)	1,223 (<1%)	485 (<1%)
Fluvoxamine maleate	1,249 (<1%)	1,026 (<1%)	223 (<1%)
Doxepin	1,166 (<1%)	785 (<1%)	381 (<1%)
Phenelzine sulfate	1,086 (<1%)	494 (<1%)	592 (<1%)
Moclobemide	758 (<1%)	395 (<1%)	363 (<1%)
Reboxetine	419 (<1%)	134 (<1%)	285 (<1%)
Mianserin	204 (<1%)	68 (<1%)	136 (<1%)
Tranylcypromine sulfate	192 (<1%)	150 (<1%)	42 (<1%)
Isocarboxazid	176 (<1%)	118 (<1%)	58 (<1%)
Naltrexone/bupropion	37 (<1%)	13 (<1%)	24 (<1%)

Table A6: PHQ9 results

PHQ9 Categorical result	Total cohort (n=31,215)	MDD patients (n=28,231)	TRD patients			MHS (n=11,360)
			All (n=2,984)	3+ lines (n=1,045)	4+lines (n=317)	
No depression	2,823	2,658	165	44	11	888
Mild depression	4,139	3,797	342	119	32	1,420
Moderate depression	6,238	5,712	526	159	42	2,108
Moderately severe depression	8,312	7,503	809	269	83	2,847
Severe depression	7,977	7,025	952	368	119	3,473
Unknown	1,726	1,536	190	86	30	624

Abbreviations: MDD, major depressive disorder; MHS, contact with secondary mental health services; PHQ9, brief Patient Health Questionnaire; TRD, treatment-resistant depressions; TRD3+, three or more lines of treatment for depression; TRD4+, four or more lines of treatment for depression.
